# Intestinal microbiota profiles in a genetic model of colon tumorigenesis correlates with colon cancer biomarkers

**DOI:** 10.1038/s41598-022-05249-0

**Published:** 2022-01-26

**Authors:** Francesco Vitali, Katia Tortora, Monica Di Paola, Gianluca Bartolucci, Marta Menicatti, Carlotta De Filippo, Giovanna Caderni

**Affiliations:** 1 Institute of Agricultural Biology and Biotechnology, National Research Council (CNR), Via Moruzzi, 1, 56124 Pisa, Italy; 2 NEUROFARBA Department, Pharmacology and Toxicology Section, University of Florence, Viale Pieraccini 6, 50139 Florence, Italy; 3 Gastroenterology and Nutrition Unit, Meyer Children’s Hospital, Florence, Italy

**Keywords:** Cancer models, Colorectal cancer, Metagenomics, Microbiology, Microbial communities

## Abstract

Faecal (FM) and colon mucosal associated microbiota (MAM) were studied in a model of colorectal cancer (CRC), the *Apc*-mutated Pirc rats, and in age-paired wt F344 rats. Principal Coordinates Analysis indicated that samples’ distribution was driven by age, with samples of young rats (1 month old; without tumours) separated from older ones (11-month-old; bearing tumours). Diversity analysis showed significant differences between FM and MAM in older Pirc rats, and between MAM of both Pirc and wt rats and the tumour microbiota, enriched in *Enterococcus, Escherichia/Shigella, Proteus* and *Bifidobacteriaceae*. In young animals, Pirc FM was enriched in the genus *Delftia*, while wt FM was enriched in *Lactobacillus* and *Streptococcus.* Some CRC biomarkers and faecal short chain fatty acids (SCFAs) were also measured. Colon proliferation and DClK1 expression, a pro-survival mucosal marker, were higher in Pirc than in wt rats, while the mucin MUC2, was lower in Pirc rats. Branched SCFAs were higher in Pirc than in wt animals. By Spearman analysis CRC biomarkers correlated with FM (in both young and old rats) and with MAM (in young rats), suggesting a specific relationship between the gut microbiota profile and these functional mucosal parameters deserving further investigation.

## Introduction

Epidemiological and experimental studies show that variation in the composition of the intestinal microbiota affects colorectal cancer (CRC) risk, accordingly, a number of studies indicates that the faecal microbiota of colon cancer patients differs from that of healthy subjects^1–4^. Moreover, not only the faecal microbiota, but also the bacterial community associated with the colon mucosa may be an important determinant of CRC risk^5,6^. In fact, it has been shown that bacterial biofilms associated with the surface of colon epithelium are increased in a subset of sporadic CRC^7^ and in the apparently normal colon mucosa of patients with familial adenomatous polyposis (FAP), a genetic syndrome causing high risk of developing CRC^5^. These biofilms contain species subtypes of *Escherichia coli* and *Bacteroides fragilis*, encoding genes for oncotoxins (colibactin) and *B. fragilis* toxin (bft).

In humans, mutations in the *APC* gene, responsible for the inherited predisposition to intestinal carcinogenesis in FAP patients and present in the majority of sporadic CRC (i.e., developing without apparent familiarity), are considered early, necessary events in the development of the disease^8^. Accordingly, rodent models mutated in *Apc* gene have been widely used to study the mechanisms of colon carcinogenesis, but also the effect of drugs or environmental factors, such as diet. Pirc rats (F344/NTac-Apc^am1137^) carry a heterozygous germinal mutation in *Apc* leading to the spontaneous development of tumours along the intestine, notably in the colon^9,10^, thus representing a relevant experimental model to study the process of colon tumorigenesis including the influence of external factors or individual host factors, such as the intestinal microbiota^11^.

Previous studies on the intestinal bacterial community in *Apc*-mutated rodents gave conflicting results. Son et al. reported that the microbiota associated to the intestinal mucosa of *Apc*-mutated *Min* mice was enriched with Bacteroidetes spp. compared with wt animals^6^. Similarly, we recently documented that the bacterial community associated with the mucosa of Pirc rats presents some differences when compared with that of wt rats^12^, thus suggesting variations in animals at higher CRC risk. On the contrary, Ericsson et al. documented that the faecal microbiota of Pirc rats and wt rats are similar^13^.

Given these contrasting results and the importance to characterize Pirc rats as a relevant model of CRC, we though it of interest to study the composition of their intestinal bacterial community in both the faeces and the colon mucosa. For this purpose, we compared the microbiota profiles of Pirc and wt rats at two different ages: 1 month-old, when the colon mucosa is morphologically normal with no macroscopic lesions^10^, and in older rats (11-month-old), when colon tumours are present. In addition, since microbiota may affect cancer risk with various mechanisms such as the production of metabolites in the intestinal lumen, in the same rats we also studied faecal short chain fatty acids (SCFAs), deriving from gut microbial fermentation of indigestible fibres and other substrates. SCFAs contribute to the homeostasis of colonocytes^14^ and were not studied before in Pirc rats. Moreover, we also determined some mucosal parameters that have been associated with CRC risk and used as cancer biomarkers. MUC2 is the main mucin secreted by colonocytes, whose alterations have been associated with an increased CRC risk^15,16^; CD68, is a pan-macrophage marker used as an index of inflammation, a phenomenon associated with high CRC risk. Since *Apc* mutations have been reported to affect colon proliferation and cell survival^8^, we also studied *PCNA* expression, as a marker of proliferation, together with *DClK1* expression, a microtubule-associated protein kinase regulating pro-survival signalling^17,18^ .

Finally, to have a glimpse on the composite relations between the faecal and mucosal microbiota, SCFAs as its produced metabolites, and the cancer biomarkers measured in the mucosa, the data obtained were analysed with a multivariate approach (i.e., Mantel -test on Spearman correlation) to look for a correlation between all these different parameters.

## Materials and methods

### Animal-sacrifice-sampling

Pirc rats (F344/NTac-Apcam1137) and wild type (wt) Fisher F344 rats were originally obtained by the National Institutes of Health (NIH), Rat Resource and Research Center (RRRC) (University of Missouri, Columbia, MO, USA) and bred in CESAL (Housing Center for Experimental Animals of the University of Florence, Italy). The colony is maintained by mating heterozygous Pirc rats with wt and pups genotyped at 3 weeks of age^9^. Twenty-six rats (13 wt and 13 Pirc) were maintained in polyethylene cages with their respective mothers up to 4 weeks of age (1 month, T1), then separated and fed with Teklad Global Rodents Diet® (ENVIGO) until 11 months of age. Rats were euthanized at one month (T1: 7 Pirc rats and 8 wt rats) and eleven months (T11: 6 Pirc rats and 5 wt rats) of age and the entire colon and small intestine opened to check for the presence of macroscopic tumours, which were counted and the diameter measured (Supplementary Table S1 summarizes the characteristics of the samples under study). For microbiota analysis, we collected faecal pellets at different times (T1; T11) a piece of normal mucosa (NM) and one tumour/animal.

The ethical policy of the University of Florence complies with the Guide for the Care and Use of Laboratory Animals of the Italian Ministry of Health (in accordance with EU Directive 2010/63/EU). Formal approval to conduct the experiments was obtained from the Animal Subjects Review Board of the University of Florence. Experiments have been reported according to ARRIVE guidelines. All experiments were in accordance with the Commission for Animal Experimentation of the Italian Ministry of Health and with EU Directive 2010/63/EU for animal experiments.

### Bacterial DNA extraction, 16S rRNA gene sequencing, and sequencing data analysis

Bacterial DNA extraction, sequencing, and sequencing data analysis were performed according to a recent work from our group^12^. Briefly, faecal pellets, colon mucosa and tumour samples were collected in RNAlater (Qiagen, Hilden, Germany) and stored at − 80 °C until DNA extraction with DNeasy PowerLyzer PowerSoil Kit (Qiagen, Hilden, Germany). Library preparation and sequencing of the hypervariable region V3–V4 of the 16S rRNA gene were performed by using an Illumina MiSeq platform with a 300-bp paired-end reads protocol. The obtained reads were pre-processed with CUTADAPT^19^ to remove primers and Illumina adapters, while SICKLE was used to remove low quality portions of the reads^20^. OTUs/ASVs identification was performed in MICCA (ver. 1.7.2)^21^ with *miccaotu* command, using the UNOISE3 algorithm^22^, while taxonomy was assigned using the RDP classifier (ver 2.11)^23^ against the RDP database.

The datasets generated during and/or analysed during the current study are available in the ENA repository, under accession number PRJEB40657 at https://www.ebi.ac.uk/ena/browser/view/PRJEB40657.

### Evaluation of proliferation, apoptosis, CD86 and DClK1 in colon mucosa and tumours

Proliferative activity in the normal colon mucosa (NM) and in tumours was evaluated measuring Proliferating Cell Nuclear Antigen (PCNA) immunoreactivity with a mouse monoclonal antibody (PC-10, Santa Cruz, CA, USA), as described^10^. CD-68 and DClK1 expressions were also evaluated in NM and tumours with immunohistochemistry experiments as reported^23–25^. Apoptosis was evaluated in histological longitudinal sections (4 µm thick) of the NM and tumours stained with hematoxylin–eosin, as reported^10^.

### MUC-2 expression in colon mucosa

MUC2 protein levels were measured by dot blot assay. Proteins were extracted from scraped NM samples harvested at sacrifice from the proximal colon and kept at − 80 °C. Samples were homogenized in RIPA-buffer containing 1% protease cocktail inhibitors and 1% phosphatase inhibitors (Sigma Aldrich, Milan, Italy), sonicated for 15 s and centrifuged at 1.000×*g* for 15 min at 4 °C. Protein content of supernatants was measured accordingly to DC Protein Assay kit instructions (Bio-Rad, Segrate-Milan, Italy). Equal volumes of supernatants containing 30 μg of proteins were allowed to dry on nitrocellulose membrane (Invitrogen) for 30 min and then washed under gentle agitation at RT for 1 h with 0.05% Tween-PBS solution (T-PBS) containing 5% dry milk (Bio-Rad, Segrate-Milan, Italy). Membranes were incubated for 1 h with 1:1000 dilution of anti-MUC2 antibody (sc-15334 Santa Cruz Biotech.), then washed with T-PBS and incubated for another 1 h with anti-rabbit IgG HRP-conjugated antibody (#7074 Cell Signalling). Finally, protein spots were visualized using the enhanced chemiluminescence procedure with Immobilon Horseradish Peroxidase Substrate (Millipore) and quantified by densitometric analysis using the Quantity-One software (Bio-Rad).

### Faecal water preparation and SCFAs quantification

#### Sample preparation

Faeces were freshly collected and stored at − 80 °C. At use, each sample was thawed and weighted (weight range 500–800 mg); then added sodium bicarbonate 10 mM (1:1 w/v) in a 1.5 mL centrifuge tube. The obtained suspension was then mixed with the aid of a sterile wooden stick and briefly shaken in a vortex apparatus, extracted in ultrasonic bath (15 min) and then centrifuged at 4 °C at 13.000 rpm for 90 min. The supernatant was collected, transferred in 1.5 mL centrifuge tube and stored at − 20 °C until use.

For the analysis, these supernatant samples were thawed, briefly centrifuged at 5000 rpm and resuspended for 5 min in ultrasonic bath. The SCFAs were then extracted as follows: an aliquot of 100 μL of sample solution (corresponding to 0.1 mg of stool sample) was added with 10 μL of internal standard (ISTD) mixture, 1 mL of tert-butyl-methyl ether and 50 μL of 1.0 M HCl solution in 1.5 mL centrifuge tube. Afterwards, each tube was shaken in a vortex apparatus for 2 min, centrifuged at 10,000 rpm for 5 min; the solvent layer was finally transferred in auto-sampler vial and analysed by Gas-Chromatography–Mass Spectrometry (GC–MS) method, using an Agilent GC–MS system composed with 5971 single quadrupole mass spectrometer, 5890 gas-chromatograph and 7673 autosampler. Details of the method are described in Supplementary Materials.

### Statistical data analysis

Statistical analysis of the microbiota data was performed using R Software as described^26^. Sequencing count data were parsed into R with *phyloseq*^27^, and prior to further analysis, reads counts were transformed with CSS (Cumulative Sum Scaling) followed by logarithm transformation as implemented in *metagenomeSeq* package^28^. Alpha diversity in bacterial communities was explored using three indices: Species Richness (i.e., the number of different OTUs in a sample), Evenness index (i.e., the grade of equitability in the distribution of relative abundances of the OTUs in a sample), and Shannon index (i.e., a measure of diversity of the community in a sample). Differences in alpha diversity indices between samples categories, were assessed with T-test. OTUs distribution in the mucosal and tumour adherent microbiota community was further explored with rank-abundance curves, obtained with the package *BiodiversityR*^29^. Beta diversity of bacterial communities was explored and visualized using Principal Coordinate Analysis (PCoA) ordinations based on the Bray–Curtis dissimilarity, with the *phyloseq* package. Associations of bacterial community diversity with experimental factors (i.e., age, rat genotype, and sample type) were assessed with permutational analysis of variance (PERMANOVA: 9999 permutations, Bray–Curtis dissimilarity) in the *vegan* package^30^, and with *Hierarchical Clustering on Principal Components* (HCPC) analysis in the *FactoMineR* package^31^. In HCPC, dimensionality of microbiota data is first reduced with the aid of principal component analysis, and then hierarchical clustering is applied. LEfSe (Linear discriminant analysis Effect Size) analysis^32^ on the CSS transformed abundances, was performed using an online Galaxy implementation (https://huttenhower.sph.harvard.edu/galaxy/) to identify plausible bacterial biomarker(s) able to separate different groups (i.e., according to different genotypes or different samples types). Significance threshold for Kruskal–Wallis rank-sum test was 0.05, and threshold for LDA (linear discriminant analysis) was 2. Data from the SCFAs analysis, and from the determination of mucosal biomarkers (PCNA, CD-68, DClK1, MUC2 expression and apoptosis) were presented as means ± SE; differences between Pirc and wt rats and between samples at T1 and T11 were analysed with two–way ANOVA considering the effect of genotype and age. All analyses were carried out using GraphPad Prism 5.0 and STATGRAPHICS Centurion XVI.II. p values of ≤ 0.05 were considered statistically significant. Mantel test was used to assess relations between the structure of the bacterial community and the measured variables (i.e., SCFAs and functional parameters in each individual rat sample) as previously described^33,34^.

## Results

### Diversity analysis of faecal and mucosal bacterial community of Pirc rats and wt rats

**Figure 1:**
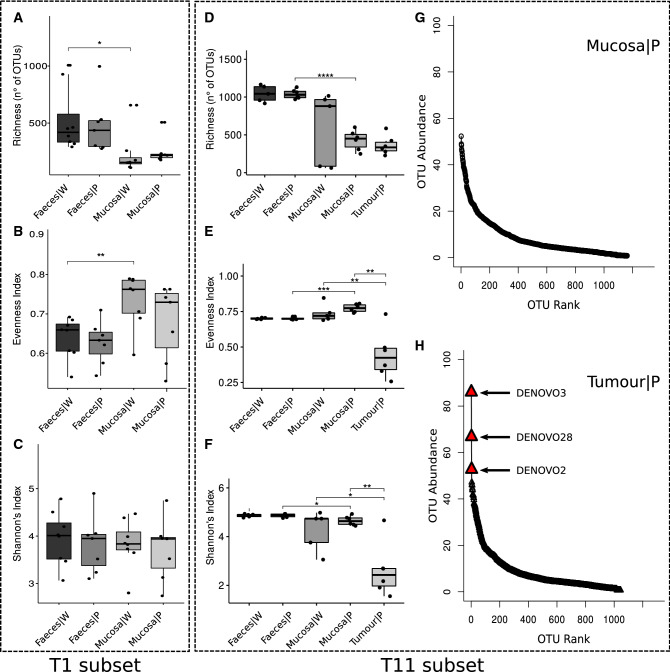
Diversity analysis of bacterial communities in rats, calculated by number of observed OTUs, Pielou’s Evenness, Shannon and Simpson indices. (**A**) Differences in observed Richness between samples groups at T1 assessed with t-test (*p-value < 0.05). (**B**) Differences in Evenness diversity index between samples groups at T1 assessed with t-test (**p-value < 0.01). (**C**) Differences in Shannon’s diversity index between samples groups at T1 assessed with t-test. (**D**) Differences in observed Richness between samples groups at T11 assessed with t-test (****p-value < 0.0001). (**E**) Differences in Evenness diversity index between samples groups at T11 assessed with t-test. (**p-value < 0.01; ***p-value < 0.001). (**F**) Differences in Shannon’s diversity index between samples groups at T11 assessed with t-test (*p-value < 0.05; **p-value < 0.01). (**G**) Rank abundance curves of mucosal samples at T11 in Pirc rats. (**H**) Rank abundance curves of tumour samples at T11 in Pirc rats.

To evaluate differences in bacterial communities among faecal and mucosal samples at different time points in the two genotypes, analyses of species richness and biodiversity were performed. In young rats (T1), by alpha diversity estimation (Fig. 1A–C), significant differences in richness (Fig. 1A) and microbial community evenness (Fig. 1B) were found between faeces and colonic mucosa in wt rats. Significant differences between Pirc and wt rats were not found, neither considering faeces nor considering mucosal microbiota. In older rats (T11) (Fig. 1D–F), differences in microbial richness between faeces and colonic mucosa were observed in Pirc rats (Fig. 1D), but not in wt animals. Considering Evenness and Shannon indexes (Fig. 1E,F), significant differences were found between mucosal microbiota and faeces of Pirc rats, as well as between microbiota of normal mucosa of both Pirc and wt rats respect to the microbiota adherent to tumours. To note, the tumour adherent bacterial community had the lowest Evenness and Shannon’s index values (Fig. 1E,F), indicating a dominance of specific bacteria in tumour tissues. Accordingly, rank abundance curves analysis, showed the dominance of few bacterial OTUs in the tumour community compared with the normal mucosa (Fig. 1G,H); in particular the three bacterial OTUs dominating over the rest of the community correspond to *Escherichia/Shigella*, *Streptococcus*, and *Bacteroides* genera, in decreasing order of dominance and relative abundance, respectively (see Table S4 in Supplementary Material Results).

**Figure 2:**
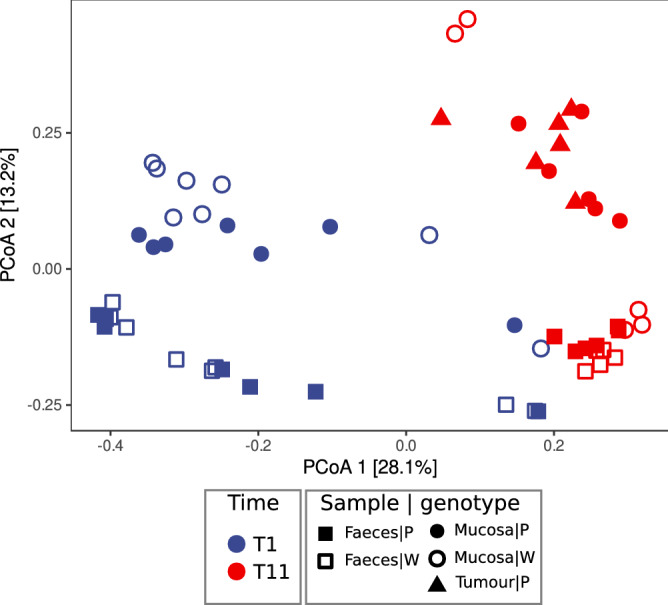
PCoA ordinations based on Bray–Curtis dissimilarity index. Ordination of all samples at T1 and T11. Shape of points indicates sample type: squares for faeces, circle for mucosa, and triangles for tumour. Empty shapes indicate wt samples, while solid/full shapes indicate Pirc samples; colour of points represents time (blue for T1 and red for T11).

To evaluate the variability of microbial communities among samples (beta diversity), PCoA based on Bray–Curtis distances was performed (Fig. 2). PCoA ordination showed that samples’ distribution of both Pirc and wt rats was mainly driven by the time point of sample collection, that is, by the age of the animals (Fig. 2), with samples of younger rats (T1) clearly separated by that of older ones (T11). This observation was further supported by PERMANOVA analysis (Table 1) in which the “age” variable was found at the highest R^2^ value (R^2^ = 0.219, p-value = 0.0001). Furthermore, on the second ordination axis (Fig. 2) discrete sample clusters on the basis of sample type (faeces, mucosa, and tumour) can be observed, as also highlighted by PERMANOVA analysis (Table 1, R^2^ = 0.11261, p-value = 0.0001). Moreover, PERMANOVA performed on T1 or T11 samples separately (Table 1), indicates that microbial communities in younger rats’ samples significantly varied only based on sample type (faeces or mucosa), while in older rats both sample type and genotype had a significant effect on the microbiota. Hierarchical Clustering on Principal Components (HCPC) analysis confirmed the above observations (see Fig. S1 in Supplementary Material Results).

**Table 1:** Results of PERMANOVA analysis to test the effects of different sources of variation (age, genotype, and sample type) on the Bray–Curtis distance matrix between all pairs of samples (T1 and T11 together), between pairs of samples at T1 (young rats), or between pairs of samples at T11 (old rats).

Source of variation	R^2^	p value
**All samples**		
Age	0.2193	**0.0001**
Genotype	0.0198	0.0839
Sample type	0.1126	**0.0001**
Interaction: age and genotype	0.0175	0.1310
Interaction: age and sample type	0.0218	0.0581
Interaction: genotype and sample type	0.0070	0.9010
Interaction: age, genotype and sample	0.0051	0.9938
Residual	0.5967	
**Only T1 samples**		
Genotype	0.0278	0.5019
Sample type	0.1484	**0.0002**
Interaction: genotype and sample type	0.0108	0.9945
Residuals	0.8130	
**Only T11 samples**		
Genotype	0.0865	**0.0041**
Sample type	0.1858	**0.0003**
Interaction: genotype and sample type	0.0213	0.7984
Residuals	0.7064	

Statistically significant values (p < 0.05) are highlighted in bold.

### Taxonomic structure of the bacterial community

**Figure 3:**
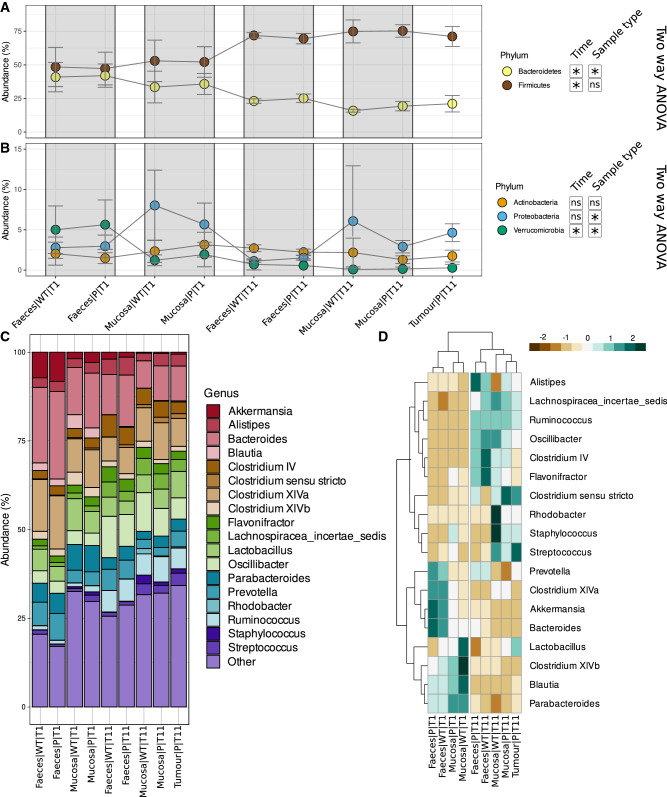
Relative abundances of phyla and genera (only phyla and genera with abundance > 5% in at least one sample were represented). (**A,B**) Composition of bacterial communities at the phylum level, grouped for sample type (faeces-mucosa) and genotype (Pirc-wt). Gray rectangles highlight couples of Pirc and wt samples from the same time point. Two-way ANOVA testing for time and genotype or for sample and genotype: *Significant effect of time (age) or sample type (faeces vs mucosa); *ns* non significant; (**C**) composition of bacterial communities at the genus level, grouped for sample type and genotype groups. Genera with relative abundance lower than 5% were grouped in the “Other” category. (**D**) Heatmap highlighting abundance distribution of genera (only genera at abundance higher than 5% were retained) among sample type and genotype groups.

Bacterial community composition (Fig. 3A) shows that Firmicutes was the dominant phylum in the overall sample categories (ranging from a mean relative abundance of 47.2% ± 12.2 (SD) in Pirc faeces at T1, to a mean of 75.2% ± 4.66 (SD) in Pirc mucosa at T11), with a marked increase from T1 to T11 (Fig. 3A). Bacteroidetes was the second most abundant phylum (ranging from a mean of 15.85% ± 1.06 SD in wt mucosa at T11 to a mean of 41.96% ± 8.68 SD in Pirc faeces at T1). Among less represented phyla (Fig. 3B), Actinobacteria was almost equally abundant among all sample types and no differences by rat aging or genotype were observed. Phylum Proteobacteria was more abundant in mucosal microbiota than in faecal one. Two-way ANOVA test (with genotype and age as test factors) confirmed that the abundance of Bacteroidetes, Firmicutes and Verrucomicrobia phyla significantly changed with rat ageing (p-values < 0.0001), while two-way ANOVA (with genotype and sample type as test factors) highlighted that Bacteroidetes, Proteobacteria, and Verrucomicrobia relative abundance changed significantly with sample type (p-values = 0.019, 0.0001, and 0.0007 respectively).

At genus level (Fig. 3C), the most representative 18 bacterial genera in wt and Pirc rats were identified in all sample types. Among these, *Bacteroides* and *Clostridium XIVa* were the most abundant genera in almost all samples, with highest relative abundances in faecal samples at T1. On the contrary, *Oscillibacter* was less abundant at T1, and increased at T11 in all samples, with exception of tumours. In general, as an effect of temporal variation (from T1 to T11), the relative abundances of *Ruminococcus, Oscillibacter, Streptococcus,* and Lachnospiraceae increase with rats’ aging, while those of *Bacteroides, Clostridium XIVa,* and *Parabacteroides* decrease (Fig. 3C). To note, at T1, in young rats, *Blautia* was generally found in faeces and colonic mucosa of both Pirc and wt rats, and at very low abundances in all T11 samples. Moreover, *Rhodobacter* was mainly found in colonic mucosa samples of wt rats at T11, and depleted in mucosa and Tumours of Pirc rats.

Heatmap analysis (Fig. 3D) on these data shows that at T1, a cluster of genera including *Bacteroides, Akkermansia, Clostridium XIVa,* and *Prevotella* was mainly associated with the faecal microbial community, with no evident differences between Pirc and wt rats. On the other hand, the cluster including *Parabacteroides, Blautia, Clostridium XIVb,* and *Lactobacillus* characterized the mucosal bacterial community, with *Blautia, Clostridium XIVb,* and *Lactobacillus* more abundant in wt mucosa than in Pirc one. The cluster of bacterial genera associated with samples collected at T11 showed minor differences in terms of abundance between faecal and mucosal communities. *Clostridium *sensu stricto was found abundant in mucosa and tumours of Pirc rats, while *Rhodobacter* and *Staphylococcus* in mucosa of wt rats. *Lactobacillus* genus appeared enriched in tumour adherent microbiota, respect to other T11 samples (Fig. 3D). At the OTU level, single OTUs (n = 22) belonging to *Lactobacillus* genus showed a differential distribution between sample groups; however, none of those OTUs were exclusively associated to tumour adherent microbiota, but these were also present in mucosa microbiota (for both wt and Pirc rats).

### Bacterial biomarker discovery and core microbiota in Pirc and wt rats

**Figure 4:**
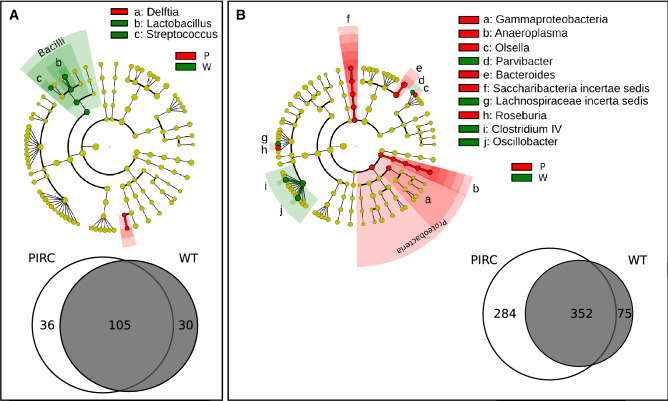
LEfSe and “core microbiota” on faecal bacterial communities at T1 (**A**) and T11 (**B**) Cladogram show the most discriminative bacterial clades. Coloured regions/branches indicate differences in the bacterial population structure between the different genotypes (Pirc in red and wt in green). Statistically significant taxa enrichment among groups was obtained with Kruskal–Wallis test among classes (Alpha value = 0.05). The threshold for the logarithmic LDA score was 2.0.

**Figure 5:**
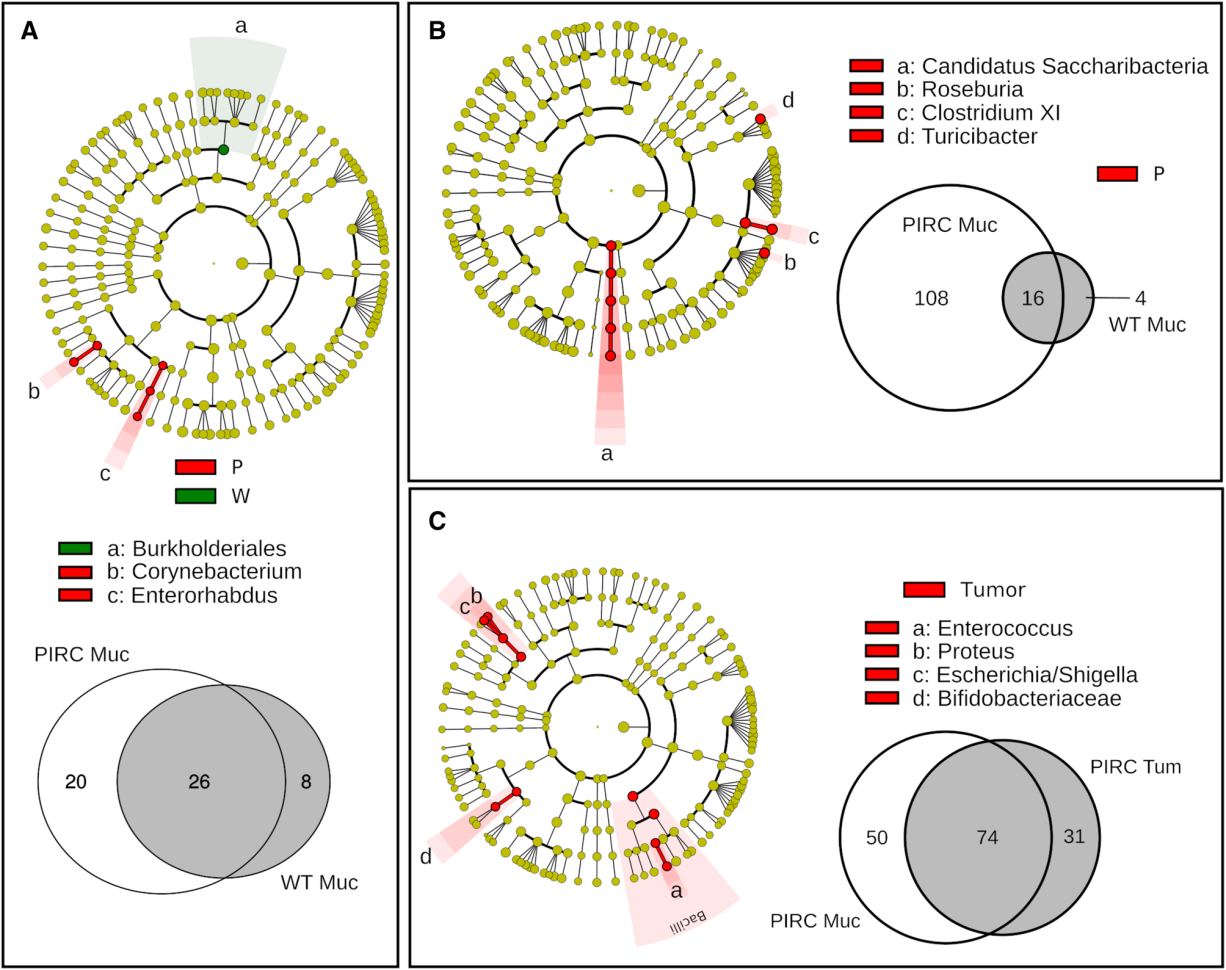
LEfSe and “core microbiota” on mucosa adherent bacterial communities at T1 (**A**) and T11 (**B**), and in comparison with tumour adherent bacterial communities (**C**). Cladograms show the most discriminative bacterial clades. Coloured regions/branches indicate differences in the bacterial population structure between the different genotypes (Pirc and wt, (**A,B**)) and between the different tissues in Pirc rats (Mucosa vs Tumour, (**C**)). Statistically significant taxa enrichment among groups was obtained with Kruskal–Wallis test among classes (Alpha value = 0.05). The threshold for the logarithmic LDA score was 2.0.

LEfSe analysis identified some microbial biomarkers characterizing the different genotypes in faecal (Fig. 4A) and mucosa samples collected at T1 (Fig. 5A). In detail, at genus level, *Delftia* was enriched in the faecal bacterial community of Pirc rats, while *Lactobacillus* and *Streptococcus* in the faecal bacterial community of wt animals (Fig. 4A). For the mucosal bacterial communities (Fig. 5A), *Corynebacterium* and *Enterorhabdus* were found enriched in Pirc, while *Burkholderiales* order in wt rats. The relative abundance of genera identified by LEfSe analysis in each individual sample is reported in the results section of Supplementary Materials (Figs. S3, S4 of Supplementary Material Results).

In addition, by comparison of the “core microbiota” (i.e., the OTUs found in 80% of the samples) from faecal samples in young rats, 105 OTUs were shared between Pirc and wt core microbiota, while a comparable number of exclusive “core OTUs” were found for each genotype (exclusive 36 OTUs for Pirc and 30 OTUs for wt; Fig. 4A, Supplementary Table S3). For mucosa samples, as expected (Fig. 5A), the number of “core microbiota” was lower than in faeces, in line with the fact that the adherent microbiota is more simplified respect to the faecal one. A total of 26 core OTUs were shared at T1 in both genotypes, while 20 OTUs were exclusively found in Pirc rats, and 8 in the wt core (Fig. 5A, Supplementary Table S4).

In older animals (T11), comparing faecal samples of Pirc and wt rats, a higher number of differences was found (Fig. 4B). Gammaproteobacteria phylum, *Anaeroplasma, Olsella, Bacteroides, Saccharibacteria incertae sedis* and *Roseburia* genera were discriminant taxa of Pirc rats, while *Parvibacter, Lachnospiraceae incertae sedis, Clostridium IV* and *Oscillibacter* were enriched in wt rats. Regarding the “core microbiota” in faecal samples collected at T11 (Fig. 4B, Supplementary Table S3), 352 OTUs were found shared between Pirc and wt rats, while a substantial number of exclusive OTUs were observed in Pirc rats (N = 284) compared with wt rats (N = 75).

For mucosal samples at T11, LEfSe analysis identified specific microbial markers only in Pirc (Fig. 5B,C). Considering the normal mucosa (Fig. 5B), *Candidatus, Saccharibacteria*, *Roseburia*, *Clostridium XI*, and *Turicibacter* were enriched in Pirc rats. Interestingly, in Pirc rats, when adherent microbiota of tumours was compared with adherent microbiota of their normal mucosa (Fig. 5C), tumour tissues were enriched in *Enterococcus, Escherichia/Shigella, Proteus* and *Bifidobacteriaceae*.

Regarding the “core microbiota”, a greater number of OTUs was found in normal mucosa of Pirc (N = 108) compared to wt (N = 4), while only 16 OTUs were shared (Fig. 5B, Supplementary Table S4). By comparison of Pirc normal mucosa with tumours, we observed a relevant number of shared “core OTUs” (N = 74), as well as in healthy mucosa (N = 50) (Fig. 5C, Supplementary Table S5).

Based on the LEfSe and on the relative abundance analyses (“Results” section of Supplementary Materials) we can identify an enrichment of *Anaeroplasma*, *Bacteroides*, *Saccharibacteria*, *Roseburia*, and *Clostridium XI* in 11-month-old Pirc rats, while *Oscillibacter* and *Clostridium IV* are enriched in 11 months old wt rats.

### Faecal SCFAs and functional mucosa parameters in the colon of Pirc and wt rats

**Figure 6:**
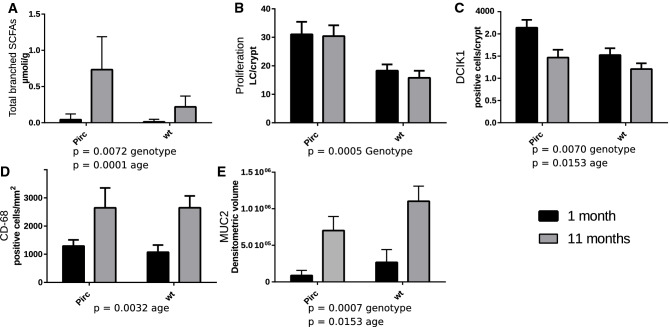
SCFAs quantification in faecal samples and mucosal functional parameters in rat colon mucosa from Pirc and wt rats sampled at T1 (black columns) or T11 (grey columns). Differences were assessed by two-way ANOVA testing for genotype and age effects, and their interaction.

SCFAs were measured in the faeces of Pirc and wt rats at the two different ages (Supplementary Table S2). Total SCFAs levels were similar among the different groups (µmoles/g faeces were: 8.37 ± 1.34, 9.86 ± 2.77, 16.5 ± 2.66 and 6.87 ± 1.07 in Pirc T1, wt T1, Pirc T11 and wt T11, respectively; data are means ± SE). No significant differences were found in the levels of acetic, propionic and butyric acids among the different groups, while for valeric acid we observed that older rats (both strains) had higher amounts than young rats. Regarding branched SCFAs (BSCFAs) as shown in Fig. 6A, we observed a significant effect of age (older rats having more BSCFAs than the younger ones) and also, a significant effect of the genotype with Pirc rats having more BSCFAs than wt rats.

Regarding functional parameters in the normal mucosa (Fig. 6), we confirmed^16^ that colon proliferative activity is significantly higher in Pirc than that in wt rats (Fig. 6B), with no effect of age in both strains. We also confirm that the level of apoptosis is similar in wt and Pirc rats (Supplementary Table S2). Furthermore, we observed that the expression of DClK1 (Fig. 6C), a putative stem-cell biomarker, was significantly higher in the crypts of Pirc rats compared with those of wt rats, and also, higher in younger than in older animals. The pan-macrophage marker CD-68 (Fig. 6D) was similar in the two strains of rats, but CD-68 expression was higher in older rats (irrespectively from strain) than in younger ones. Finally, we also found that the expression of MUC2 (Fig. 6E), was lower in Pirc rats compared with wt rats, and that its expression was significantly higher in older rats than in young ones, irrespectively from the strain of the rats. Examples of PCNA, DclK1, and CD-68 determination by immunohistochemistry experiments, can be found in Supplementary Fig. S5.

### Interrelations between microbiota composition and functional parameters in the mucosa

We finally aimed at identifying possible relationships between the microbiota present in the faeces or in the mucosa and the functional parameters that were tested. Mantel test was used to find and test the Spearman correlation between the Bray–Curtis distance matrix (calculated on the bacterial community data) and the Euclidean distance matrices of: (i) concentration of individual SCFAs (Acetic, Propionic, Butyric, Iso-Butyric, Iso-valeric, 2-Methyl-Butyric, Valeric, Hexanoic); and (ii) values of functional parameters (PCNA, CD68, DClK1, MUC2 expression and apoptosis) on the same set of samples. The test was performed on different set of samples on the basis of age and sample type (faeces and mucosa—Table 2). Regarding samples in young rats, a significant Spearman correlation was observed between mucosal microbiota profiles and the functional variables parameters (R = 0.32, p = 0.03); a borderline significant association between faecal microbiota and the functional parameters (R = 0.27, p = 0.058) was also found. In older rats, a significant correlation (R = 0.37 p = 0.03) was found between faecal microbiota and functional parameters. No other combinations resulted significant.

**Table 2:** Spearman correlation coefficients (R^2^) and significance (p-value) of mantel tests between Bacteria community structure (Bray–Curtis distance) and SCFAs (acetic, propionic, butyric, iso-butyric, iso-valeric, 2-methyl-butyric, valeric, hexanoic) or mucosal functional parameters (PCNA, CD68, DClK1, Apoptosis, MUC2).

		SCFAs Level	Functional parameters
**Only T1 samples**			
Mucosa	Functional parameters	R^2^ = 0.24	
		p = 0.09	
	Microbiota	R^2^ = 0.07	**R**^**2**^** = 0.32**
		p = 0.33	**p = 0.03**
Faeces	Functional parameters	R^2^ = 0.12	
		p = 0.28	
	Microbiota	R^2^ = 0.13	R^2^ = 0.27
		p = 0.23	p = 0.06
**Only T11 sample**			
Mucosa	Functional parameters	R^2^ = 0.12	
		p = 0.30	
	Microbiota	R^2^ = − 0.07	R^2^ = − 0.25
		p = 0.53	p = 0.90
Faeces	Functional parameters	R^2^ = 0.12	
		p = 0.29	
	Microbiota	R^2^ = 0.12	**R**^**2**^** = 0.37**
		p = 0.32	**p = 0.03**

Statistically significant values (p < 0.05) are highlighted in bold.

## Discussion

Accumulating evidences, driven by the metagenomics breakthrough during the last decade, are linking CRC to specific signatures of what could be considered an “oncogenic” microbiota^35,36^, however more efforts are still needed to gain insight in the mechanistic determinants of this association and the translation of these findings into clinical practice. Animal models represent a fundamental tool to shed light into the mechanism connecting changes in the intestinal microbiota with CRC initiation and progression. In this light, the Pirc rat model, among other *Apc*-mutated animal models (i.e., the Min mice), is of particular interest, faithfully reproducing many traits of human CRC biology, in particular the development of tumours in the colon, the very tract of the intestine more frequently affected by cancer in humans^10,11^. Notwithstanding its relevance as a model to study CRC, insight into the Pirc gut microbiota is still scarce.

In the present work we aimed at defining the structure of both the faecal and mucosal-associated microbiota in Pirc rats, along with its changes during the colorectal tumorigenesis process and its association with host functional parameters and with microbial metabolic markers.

Our results on diversity analysis of the faecal and mucosal bacterial community suggested that the gut microbiota richness and diversity were mainly influenced by the type of samples, with the faecal community richer than the adherent one but less homogeneous, especially in young animals (T1). Moreover, beta diversity analysis, revealed that the main factor shaping faecal and mucosal bacterial communities was the rat age with a low contribution from rat genotype. This result is in agreement with a previous study in Pirc rats^13^ reporting that the faecal microbial community is shaped by age (1 month and 4 months), rather than by genotype (Pirc vs wt). On the other hand, considering separately only old rats (T11), diversity analysis showed differences in bacterial community clearly related to the genotype (Table 1). Mucosal microbial community in older Pirc rats (normal mucosa and tumour alike) was in fact different compared with that found on the wt rat mucosa, and also respect to the microbiota in faeces of both wt and Pirc rats (Fig. 1D). Differences in the mucosal microbiota between Pirc and wt rats were hardly visible, and become evident only at 11 months of age. Based on this result we could speculate that the gut microbiota in young rats (1 month of age) had no time to develop, masking possible differences related to the different genotype.

Intriguingly, alpha diversity analysis highlighted a strong difference in the Evenness and Shannon’s indices of the microbiota adherent to the tumours with respect to all other samples, together with unaltered Richness compared with the normal mucosa. Three OTUs dominated the tumour microbiota which was otherwise similar to the normal mucosal microbiota from the same subject. This evidence, together with the observed similarity in beta diversity between normal mucosa and tumour adherent microbiota, suggests that the latter is dominated by a subset of the same OTUs that normally colonizes the mucosa, which, by some mechanism, expands and dominates the community in the tumour tissue. These 3 dominant OTUs were identified as belonging to the *Bacteroides, Escherichia/Shigella,* and *Streptococcus* genera, a result, that at least for*, Escherichia/Shigella* was also confirmed by the LEfSe analysis which identified this genus as a marker for the tumour microbiota compared to the normal mucosa of Pirc rats. Members of the *Escherichia/Shigella* genus such as pathogenic *E. coli*, can acquire the ability to produce genotoxins, which could be involved in the carcinogenesis process, as observed in colon cancer-associated *E. coli* strain^37^ in *Min* mice and by several reports indicating that *E. coli* frequently colonizes cancer lesions and neighbouring epithelium^37–39^.

Moreover, specific endotoxin producing strains of *Bacteroides fragilis* and *E. coli* were reported as associated to the biofilm of both the normal mucosa and the polyps in FAP patients. Besides reporting the presence of such bacterial strain, Dejea et al.^5^ demonstrated their carcinogenic effect when inoculated in pathogen-free wt mice, especially when a co-inoculation of both strains was performed. The evaluation of mucosal biofilm was beyond the scope of this work, nevertheless the observed dominance of OTUs from the *Bacteroides* and *Escherichia/Shigella* on Pirc tumours respect to normal mucosa, suggests that in Pirc rats, a similar phenomenon could be present.

LEfSe analysis indicated that the microbiota adherent to the normal mucosa of old Pirc rats (T11) was enriched in *Anaeroplasma*, *Bacteroides*, *Saccharibacteria*, *Roseburia*, and *Clostridium XI*, while *Oscillibacter* and *Clostridium IV* were enriched in 11 months old wt rats.

These finding are in agreement with recently reported significant differences between the two genotypes^12^, with Pirc rat colon mucosa enriched in *Clostridium cluster XI* (a bacterial group encompassing harmful bacteria), while the wt colon mucosa was enriched in *Clostridium clusters IV*, a bacterial group including several anti-inflammatory and butyrate-producing species^40^.

Regarding differential abundance analysis in young rats (when tumours are not present), as reported above differences between Pirc and wt rats were almost negligible with diversity analysis. However, we identified a set of possible markers of the Pirc microbiota in faeces and in mucosa which are interesting given that the absence of tumours in these animals (T1) reduces possible interferences on the microbiota composition from the peculiar tumour’s environment. Accordingly, in these young rats, *Delftia* and *Corynebacterium* genera were identified as markers of Pirc genotype in faeces and mucosa, respectively this specific finding could imply a causal relationship with tumour development since. LPS from *Delftia tsuruhatensis* can regulate cell proliferation and differentiation in murine colonic crypt-derived organoids^41^. Regarding the genus *Cory*ne*bacterium*, Zorron et al.^42^ found a higher relative abundance in colorectal invasive cancers suggesting a role of this bacterial genus in cancer progression. We also found that *Lactobacillus* and *Streptococcus* are enriched in the faecal bacterial community of wt, while in mucosal-associated bacterial community in wt rats was enriched in *Burkholderiales.*

Stepping from microbiota analysis to metabolite and mucosal functional parameters, we found that BSCFAs, mainly isovalerate, isobutyrate, and 2‐methylvalerate, were higher in Pirc genotype. BSCFAs are the bacterial fermentation products of undigested proteins and peptides reaching the colon^43,44^. The relative abundance of proteolytic bacteria such as Enterococcaceae as found in tumour samples may influence BSCFAs production^45^, leading to an increase in the production of detrimental putrefactive products such as ammonia, hydrogen sulphide and indoles^44^. We did not find other significant differences in SCFAs level in the faeces. Regarding functional mucosal parameters associated with cancer risk, we confirm a higher level of proliferation in the colon mucosa of Pirc rats, as previously documented by our group^10^. On the other hand, we did not find any difference between the two strains of rats in the expression of the pan-macrophage marker CD-68, although the expression of this marker was higher in older rats (irrespectively from strain). Although it is possible that indeed inflammation in the colon is not different between the two strains of rats, it is also possible that the macrophage accumulation in older rats overcomes any possible difference in CD-68 staining between the two strains of animals. Moreover, we should stress that although CD-68 is a commonly used marker of macrophages, including those associated with CRC, it is not able to discriminate between the different subtypes of macrophages (i.e., M1 and M2), a this fact constitutes a limitation of this study. Indeed, the phenotypic recognition of the different type of macrophages would have required the use of a double staining with additional markers, a characterization that could be deepen in future studies^46^. In addition, we showed that the expression of MUC2, the most abundant mucin secreted in the colon, was lower in Pirc rats compared with wt animals, and that this expression was significantly higher in older rats than in young ones, irrespectively from the strain of the rats. Several studies have found that MUC2 plays a crucial role in protecting the gut and maintaining intestinal homeostasis^15^. In particular, the lack of MUC2 has been related to colon cancer development in Muc2−/− deficient mice^15^. The lower expression of MUC2 in Pirc rats could be linked with their mutation in *Apc*, a genetic alteration leading to up-regulation of Wnt-signalling pathway which negatively regulates MUC2 expression^47^. Moreover, based on evidences showing that the mucus layer attenuates the cytotoxic activity of the *E. coli* phylotype B2 in vitro^48^, it is possible to speculate that the lower level of MUC2 in Pirc rats may expose their epithelium to potentially cytotoxic bacterial strains^48^, an hypothesis that should be verified in vivo*,* labelling with probes recognizing those specific bacteria the colon mucosa of both wt and Pirc rats^5^, as well of clinical specimens from patients at various CRC risk. Finally, in this work, we showed for the first time that the expression of DClK1, a microtubule-associated protein kinase identified in the intestine as a marker of tuft cells and regulating pro-survival signalling, was significantly higher in the crypts of Pirc rats compared with those of wt rats, in agreement with higher expression found in *Apc*-mutated min mice^17^.

To acquire a glimpse on the possible relations between the microbiota composition, the SCFAs as its produced metabolites, and the functional parameters of the mucosa as a consequence, we further analysed the whole data of microbiota composition, the whole set of SCFAs measured, and the whole set of functional parameters with a multivariate approach (i.e. Mantel Test on Spearman correlation). A significant Spearman correlation in Mantel test implies a similar structure in the two distance matrices compared, hence, that some kind of relation exists between the measured set of variables. Both Pirc and wt rats were included with all the data measured on the same set of animals, thus representing different aspects of their gut biology. Interestingly, in young rats, the microbial community composition in the mucosa was significantly correlated to the functional parameters measured at the same time, while the microbial communities in faeces resulted correlated with borderline significance. Thus, even if the diversity analysis alone failed to highlight a difference between Pirc and wt microbial communities’ composition in young rats, the evidence of a correlation with functional parameters, most of which presents significant differences between Pirc and wt rats (i.e., proliferation activity, DClK1, MUC2 expression), suggests that some differences are present.

## Conclusions

We performed a meta-taxonomic analysis of faecal and colon mucosal adherent microbiota in Pirc rats, mutated in the CRC key gene *Apc* and in age-paired wt rats. PCoA on the samples collected indicates that the distribution of both Pirc and wt rats was mainly driven by the age of the rats and, partially by sample type (i.e., faecal community different from that associated to the mucosa) with significant effect of the different genotype on older animals.

Differential abundance analysis identified a set of possible markers for the Pirc genotype microbiota in faeces and in colon mucosa, in particular at 11 months of age, as well as markers of microbiota adherent to tumours respect to that adherent to the normal mucosa.

We also showed that BSCFAs were higher in Pirc rats and that some functional parameters representing CRC biomarkers (proliferation activity, DClK1 and MUC2 expression) were significantly different in Pirc rats as compared to wt rats.

Finally, we found a significant correlation between the microbiota and these CRC biomarkers. Since the differences in these biomarkers are significantly different between the two strains, this result suggests a specific relationship between the microbiota profile and the colon mucosa which should be deepen studying in both pre-clinical and clinical specimens, not only the faecal microbiota but also that associated with the colon mucosa.

## Supplementary Information

Supplementary Tables.

Supplementary Information.
