# Supplementary material for: Intestinal microbiota profiles in a genetic model of colon tumorigenesis correlates with colon cancer biomarkers
Source: Sci Rep. 2022 Jan 26;12:1432. doi: 10.1038/s41598-022-05249-0 (PMC8792020; doi:10.1038/s41598-022-05249-0)
Supplement: Supplementary file 2 — Supplementary Information. [file 41598_2022_5249_MOESM2_ESM.pdf]

Supplementary Material

Intestinal microbiota profiles in a genetic model of colon tumorigenesis correlates with colon cancer biomarkers.

Francesco Vitali[1], Katia Tortora[2], Monica Di Paola[3], Gianluca Bartolucci[2], Marta Menicatti[2], Carlotta De Filippo[1*], Giovanna Caderni[2]

Francesco Vitali, ORCID 0000-0001-9125-4337

Katia Tortora, ORCID 0000-0002-0932-9983

Monica Di Paola, ORCID 0000-0002-2324-2649

Gianluca Bartolucci, ORCID 0000-0002-5631-8769

Marta Menicatti, ORCID 0000-0001-8302-7644

Carlotta De Filippo, ORCID 0000-0002-2222-6524

Giovanna Caderni, ORCID 0000-0002-0039-4738

1. Institute of Agricultural Biology and Biotechnology, National Research Council, Via Moruzzi, 1, 56124 Pisa, Italy
2. NEUROFARBA Department, Pharmacology and Toxicology Section, University of Florence, Viale Pieraccini 6, 50139 Florence, Italy;
3. Gastroenterology and Nutrition Unit, Meyer Children's Hospital, Florence, Italy.

Keywords: Pirc rat, colon carcinogenesis, gut microbiota, SCFAs, colon cancer biomarkers

*Corresponding author:
Carlotta De Filippo, PhD
Institute of Agriculture Biology and Biotechnology
National Research Council (CNR)
Via Moruzzi 1, 56124
Pisa, Italy
E-mail: carlotta.defilippo@ibba.cnr.it

SUPPLEMENTARY METHODS

Fecal water preparation and SCFAs quantification

Sample preparation

Faecal samples were collected and stored at -80 °C. Just before the analysis, each sample was thawed, weighted (between 500 mg) and added of sodium bicarbonate 10mM solution (1:1 w/v) in a 1.5 mL centrifuge tube. The obtained suspension was briefly stirred in vortex apparatus, extracted in ultrasonic bath (for 15 min) and then centrifuged at a temperature of 4°C at 13.000 rpm for 90 min. The supernatant was collected and transferred in 1.5 mL centrifuge tube (Faecal Water) and stored at -20 °C until use.

At the analysis, each Faecal Water was thawed, briefly centrifuged at 5000 RPM to spin down all the liquid, and finally resuspended for 5 minutes in ultrasonic bath. The SCFAs were finally extracted as follows: An aliquot of 100 µL of sample solution (corresponding to 0.1 mg of stool sample) was added of 10 µL of ISTDs mixture, 1 mL of tert-butyl-methyl ether and 50 µL of 1.0 M HCl solution in 1.5 mL centrifuge tube. Afterwards, each tube was shaken in vortex apparatus for 2 min, centrifuged at 10000 rpm for 5 min, and finally the solvent layer was transferred in autosampler vial and analysed by GC-MS method.

Instrumental and chemicals

The SCFAs' analysis was performed by Agilent GC-MS system composed with 5971 single quadrupole mass spectrometer, 5890 gas-chromatograph and 7673 autosampler. Methanol and tert-Butyl methyl ether (Chromasolv grade), Sodium bicarbonate and Hydrochloric acid (Reagent grade), [2H3]Acetic, [2H3]Propionic, [2H7]iso-Butyric and [2H9]iso-Valeric, used as internal standards (ISTDs), acetic acid, propionic acid, butyric acid, isobutyric acid, valeric acid and isovaleric acid (analytical standards grade) were purchased by Sigma-Aldrich (Milan, Italy). MilliQ water 18 MΩ cm was obtained from Millipore's Simplicity system (Milan, Italy).

GC-MS method

The SCFAs in the faecal water samples were analysed as free acid form using a Supelco Nukol column 30 m length, 0.25 mm internal diameter and 0.25 µm of film thickness by using the oven temperatures program as follows: initial temperature of 40 °C was held for 1 min, then it was increased to 150 °C at 30 °C/min, finally grow up to 220 °C at 20°C/min. A 1 µL aliquot of extracted sample was injected in splitless mode (splitless time 1 min) at 250 °C, while the transfer line temperature was 280 °C. The used carrier gas was helium and its flow rate maintained at 1 mL/min for whole run time. The MS acquisition was carried out in single ion monitoring by apply a proper dwell time (20 ms for each ion monitored) to guarantee a detection frequency of 4 cycle/s. The quantitative determination of SCFAs in each sample was carried out by the ratio between the area abundance of the analytes with the area abundance of respective labelled internal standard (isotopic dilution method). The value of this ratio was named peak area ratio (PAR) and it was used as abundance of each analyte in the quantitative evaluation. The ionic signals of SCFAs' and the reference internal standards used for the quantitation of each SCFAs were reported in the Table 1.

Supplementary Table 1: Retention times (Rt), ionic signals, internal standards (ISTD) for each short chain fatty acid (SCFA)

SCFA	Rt (min.)	Quan. Ion (m/z)	Qual. Ion (m/z)	ISTD [Quan. ion (m/z)]
Acetic	6.05	60	-	[2H3]Acetic [63]
Propionic	6.08	74	73	[2H3]Propionic [77]
iso-Butyric	6.22	73	88	[2H7]iso-Butyric [77]
Butyric	6.53	60	73	[2H3]Propionic [77]
iso-Valeric	7.13	60	87	[2H9]iso-Valeric [63]
Valeric	7.08	60	87	[2H9]iso-Valeric [63]

Standard solutions and calibration curves

The stock solutions of each analyte and each ISTDs were prepared in mQ water at 50 mg/mL and stored at 4 °C. Since the quantity of each SCFA in the samples could be different, different concentration ranges of each analyte were defined. Therefore, to easily build up these calibration levels, a working mixture of analytes (Mix 1) and a mixture of ISTDs in 10 mM NaHCO3 solution were prepared. The compositions and the concentrations of these mixtures are reported in the Supplementary Table 2. A five levels calibration curve was prepared by adding proper volumes of Mix 1 solution, 10 µL of ISTDs mixture, 1 mL of tert-butyl methyl ether and 50 µL of 1.0 M HCl solution in microcentrifuge tube. Then, each tube was shaken in vortex apparatus for 2 min, centrifuged at 10000 rpm for 5 min, and finally the solvent layer was transferred in autosampler vial and analysed three times by GC-MS method. Final concentrations of calibration levels are shown in the Table 2.

Supplementary Table 2: Compositions and concentrations of standard and internal standards mixtures

Acids Mixtures	Acetic (µg/mL)	Propionic (µg/mL)	Butyric (µg/mL)	iso-Butyric (µg/mL)	iso-Valeric (µg/mL)	Valeric (µg/mL)
Mix 1	1000	500	500	100	100	100
	[2H3]Acetic (µg/mL)	[2H3]Propionic (µg/mL)		[2H7]iso-Butyric (µg/mL)	[2H9]iso-Valeric (µg/mL)	
ISTDs	1000	500		100	100	

Calibration curves

Calibration curves of analytes were obtained by plotting the PAR, between quantitation ions of each analyte and relative ISTD, vs the nominal concentration of the calibration solution. A linear regression analysis was applied to obtain the best fitting function between the calibration points.

Supplementary Table 3: Compositions and concentrations of standard and internal standards mixtures

	Acetic (µg/mL)	Propionic (µg/mL)	Butyric (µg/mL)	isoButyric (µg/mL)	isoValeric (µg/mL)	Valeric (µg/mL)
1	3	1.5	1.5	0.3	0.3	0.3
2	5	2.5	2.5	0.5	0.5	0.5
3	10.0	5.0	5.0	1.0	1.0	1.0
4	20.0	12.5	12.5	2.5	2.5	2.5
5	50.0	25.0	25.0	5.0	5.0	5.0

SUPPLEMENTARY RESULTS

Rank abundance curves analysis

In the analysis reported in Figure 1G-H of the main text, OTUs in each sample type and genotype combination were ranked in decreasing order based on relative abundance (i.e. the most abundant get rank 1, the second most abundant take rank 2, and so on), and a plot of the relative abundance of each rank was produced. Higher richness values of the community produce higher maximum rank number, while the evenness values of community change the curves shapes. In an even community, the abundance is expected to decrease smoothly as the rank increases, this is what can be observed for normal mucosa adherent communities. On the contrary, in an uneven and dominated community, the decrease in relative abundance is expected to show steps, and this is what we observed for tumour adherent communities.

Based on this analysis, three bacterial OTUs dominate over the rest of the community in tumour adherent microbiota. Interestingly, those three OTUs corresponded to the *Escherichia/Shigella*, *Streptococcus*, and *Bacteroides* genera, in decreasing order of dominance and relative abundance (See Supplementary Table 1 for a list of the five-top ranked OTUs for each group, end their identification to the Genus level). Even if we were unable to further identify the species of DENOVO3, BLAST identification for DENOVO28 was *Streptococcus azizii* while it was *Bacteroides vulgatus* for DENOVO2. Those three dominant OTUs in tumour mucosa seem to be of varying importance in the community of other sample type. As for DENOVO 3 and DENOVO2, the difference is not drastic, and they are found in the Top 10 ranks of other samples group (DENOVO3: Mucosa WT at rank 7, Mucosa PIRC at rank 5; DENOVO2: Mucosa WT at rank 6, Mucosa PIRC at rank 9). The OTU DENOVO 28 is found at rank 2 in the tumour microbiota, but found at rank 82 and 88 in the mucosal microbiota of PIRC and WT, respectively.

Supplementary Table 4: Top 5 rank OTUs in the bacterial community adherent to the tumour (Pirc rats) or the normal mucosa (both Pirc and wt) at T11.

	OTU	Mean Relative Abundance in Pirc Tumour (SD)	Mean Relative Abundance in Pirc Mucosa (SD)	Mean Relative Abundance in wt Mucosa (SD)	Genus
Tumour T11 rank 1	DENOVO3	1.040 (0.263)	0.50 (0.179)	1.03 (1.22)	*Escherichia/Shigella*
Tumour T11 rank 2	DENOVO28	0.814 (0.280)	0.238 (0.133)	0.329 (0.492)	*Streptococcus*
Tumour T11 rank 3	DENOVO2	0.654 (0.306)	0.476 (0.210)	0.577 (0.405)	*Bacteroides*
Tumour T11 rank 4	DENOVO13	0.569 (0.170)	0.557 (0.142)	0.858 (0.763)	*Clostridium XlVa*
Tumour T11 rank 5	DENOVO170	0.544 (0.255)	0.077 (0.088)	0.250 (0.477)	*Proteus*
Mucosa T11 rank 1	DENOVO13	0.569 (0.170)	0.557 (0.142)	0.858 (0.763)	*Clostridium XlVa*
Mucosa T11 rank 2	DENOVO14	0.538 (0.142)	0.501 (0.052)	0.858 (0.763)	NA *(Ruminococcaceae)*
Mucosa T11 rank 3	DENOVO11	0.536 (0.144)	0.517 (0.122)	0.761 (0.721)	NA *(Porphyromonadaceae)*
Mucosa T11 rank 4	DENOVO43	0.474 (0.300)	0.535 (0.316)	0.623 (0.491)	NA *(Lachnospiraceae)*
Mucosa T11 rank 5	DENOVO3	1.040 (0.263)	0.50 (0.179)	1.03 (1.22)	*Escherichia/Shigella*
Mucosa WT T11 rank 1	DENOVO7	0.542 (0.182)	0.506 (0.234)	0.786 (0.575)	NA *(Lachnospiraceae)*
Mucosa WT T11 rank 2	DENOVO36	0.440 (0.116)	0.440 (0.116)	0.886 (0.805)	*Lachnospiraceae incertae sedis*
Mucosa WT T11 rank 3	DENOVO13	0.569 (0.170)	0.557 (0.142)	0.858 (0.763)	*Clostridium XlVa*
Mucosa WT T11 rank 4	DENOVO11	0.536 (0.144)	0.517 (0.122)	0.761 (0.721)	*NA (Porphyromonadaceae)*
Mucosa WT T11 rank 5	DENOVO5	0.491 (0.106)	0.374 (0.200)	0.523 (0.660)	*Prevotella*

HCPC clustering analysis

To strengthen the observations on the effect of age and genotype on shaping of the gut microbiota reported in figure 2 of the main text, we performed cluster analysis based on Hierarchical Clustering on Principal Components (HCPC) method. We identified four discrete clusters (Fig. 1), based on time points (clusters 1 and 3 at T1; clusters 2 and 4 at T11), genotype and sample types. For clusters 2 and 4, samples' distribution reflected genotype and sample type differences with cluster 2 including mostly mucosa and tumour samples of Pirc rats and cluster 4 with mixed sample types, such as faecal samples of both Pirc and wt and 3 wt mucosal samples, as well as 2 mucosa and one tumour of Pirc. Overall, no different distribution was observed for faecal samples among genotypes; both wt and Pirc samples were included in the cluster 4, and we can consider cluster 2 to be representative of microbiota of Pirc tissues at T11 (both mucosa and tumour), which are fairly different from wt tissues of cluster 4.

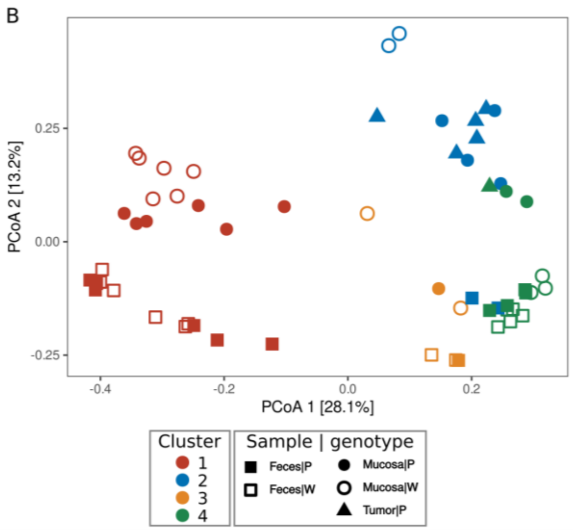

Supplementary Figure 1: PCoA ordinations based on Bray-Curtis dissimilarity index with colour of point representing results of HCPC clustering.

Family Level composition

An overview of the microbiota composition at the Family level of all samples is provided in Supplementary Figure 2. Samples were ordered on the basis of Bray-Curtis distances, and confirmed that clusters of samples were mainly grouped by the two different time points. Samples from T1 were further grouped by sample types (faeces and mucosa), while samples from T11 were grouped mainly by genotype.

Microbiota composition of the samples showing the aging process (from T1 to T11) was characterized by an almost total loss of Verrucomicrobiaceae (pink bars) and reduction of Bacteroidaceae families. This depletion was counterbalanced by an increase in Ruminococcaceae (brown bars) and Lachnospiraceae, and a decrease in Lactobacillaceae and Prevotellaceae. Overall, ANOVA analysis of the most important families (i.e., those with relative abundance higher than 5% in at least one sample; considering time point, genotype,

and sample type as analysis factors) identified a limited set of families whose relative abundance changes on the basis of those experimental factors.

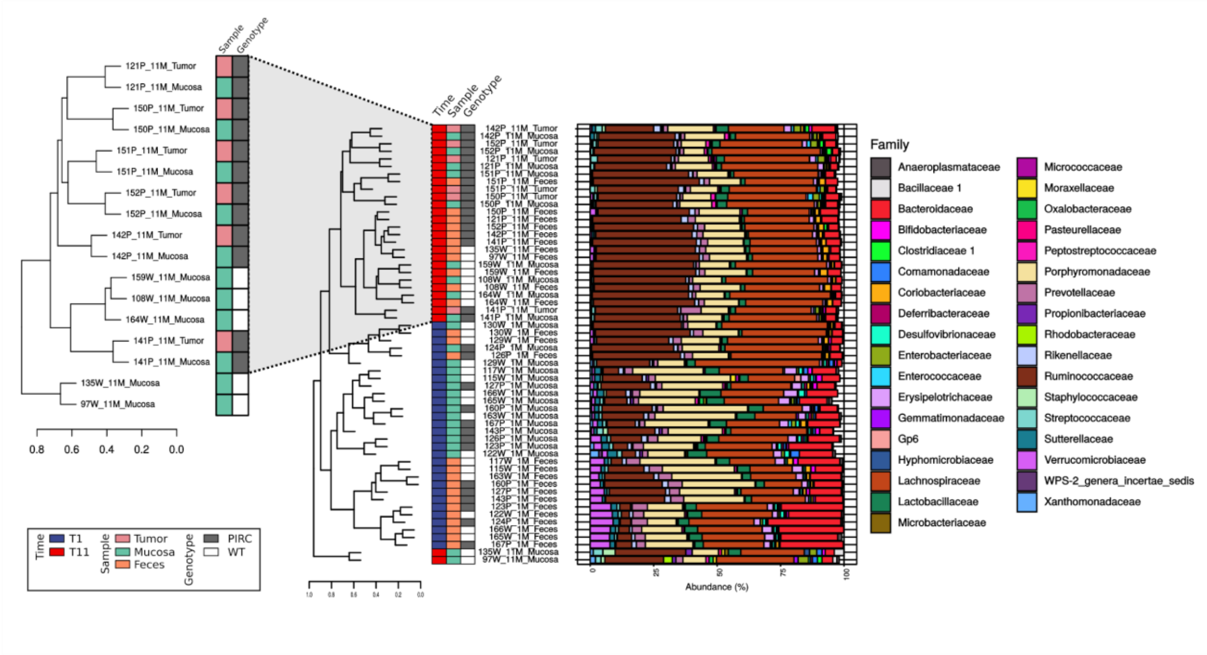

Supplementary Figure 2: Composition of bacterial community at the Family level

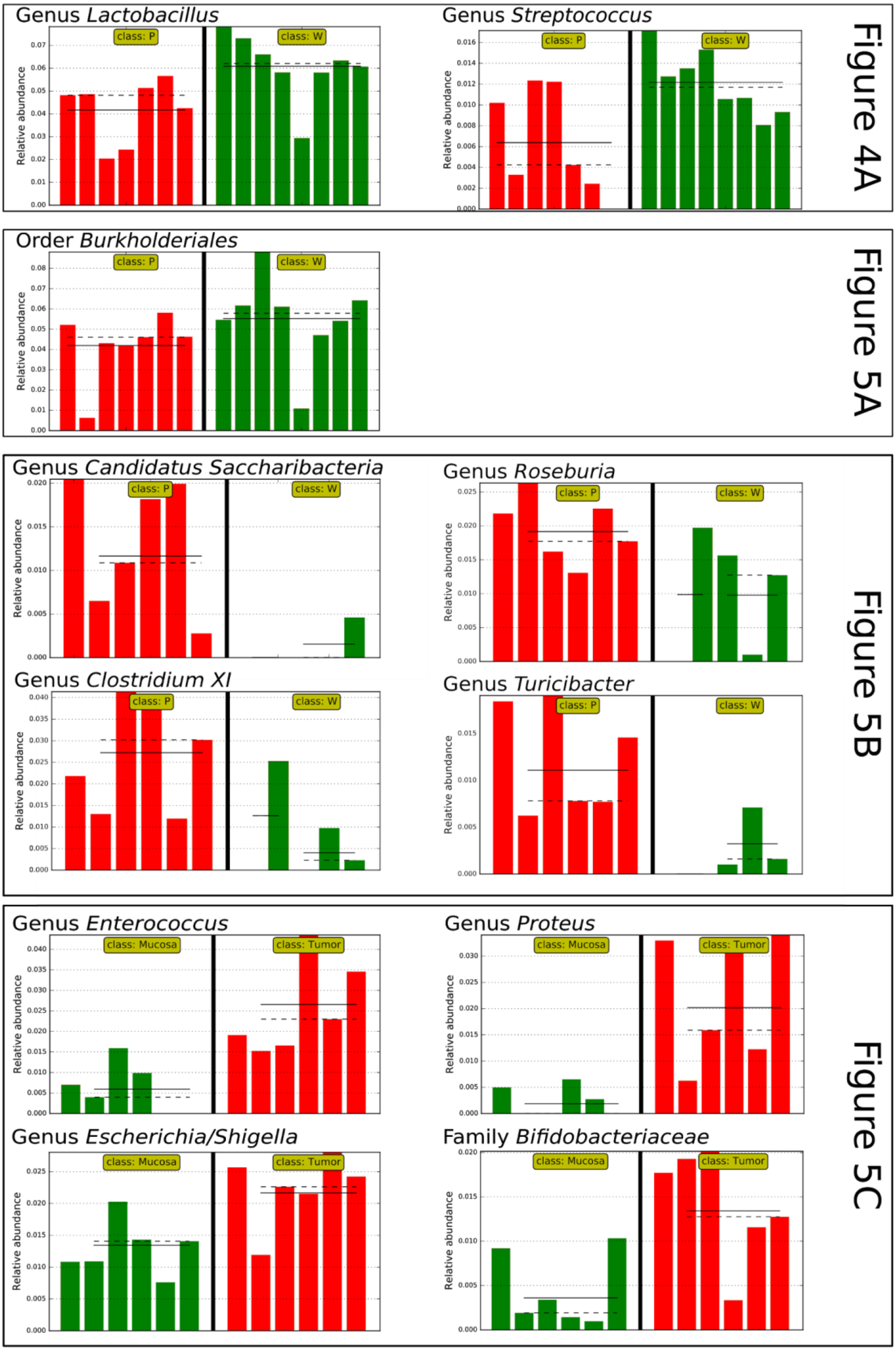

Supplementary Figure 3: Relative abundance distribution among different samples of the most promising markers in figures 4 and 5 of the main text of the manuscript. Colour of bars indicate the sample class, while solid and dashed lines represent the group mean and median, respectively

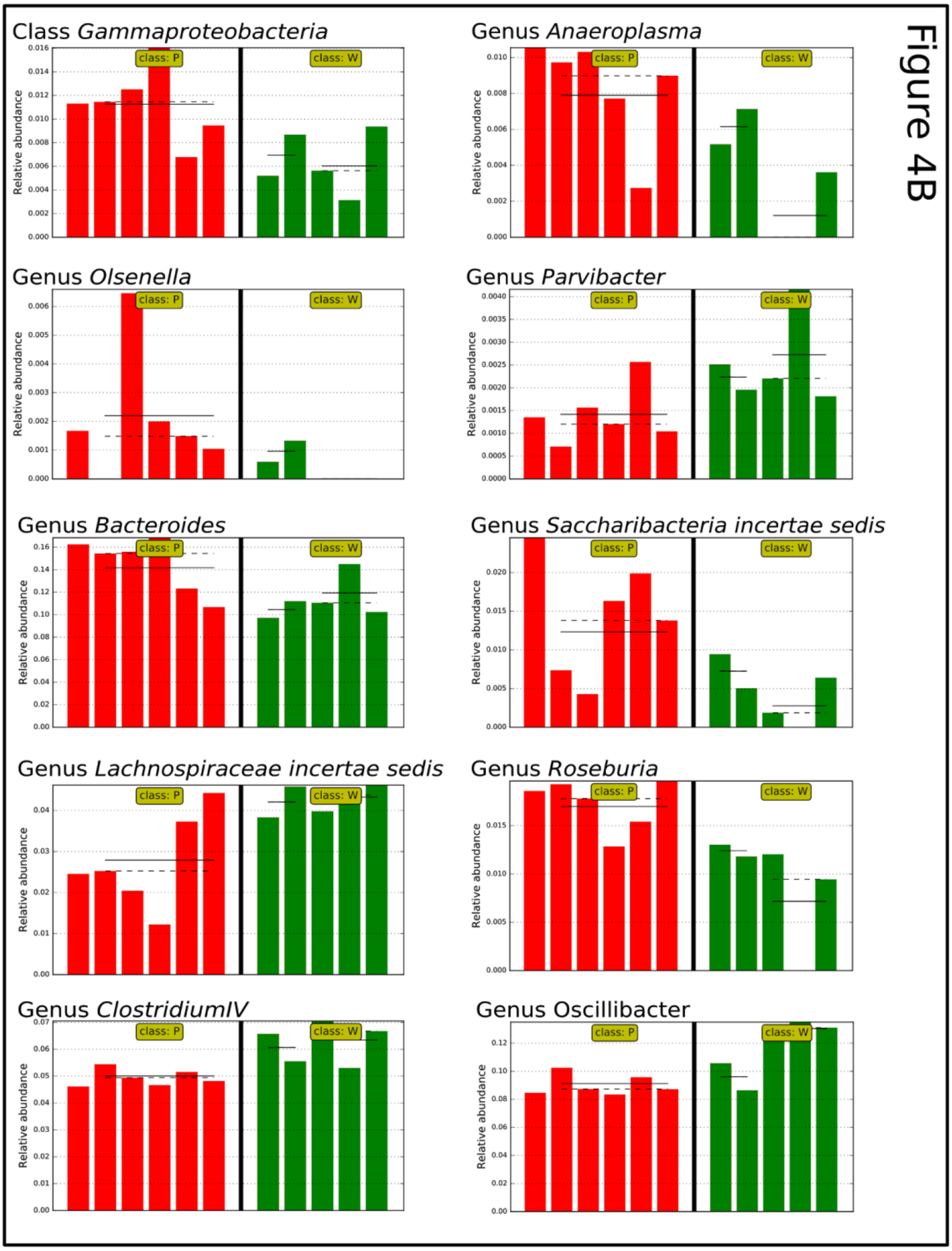

Supplementary Figure 4: Relative abundance distribution among different samples of the most promising markers in figure 4 of the main text of the manuscript. Colour of bars indicate the sample class, while solid and dashed lines represent the group mean and median, respectively

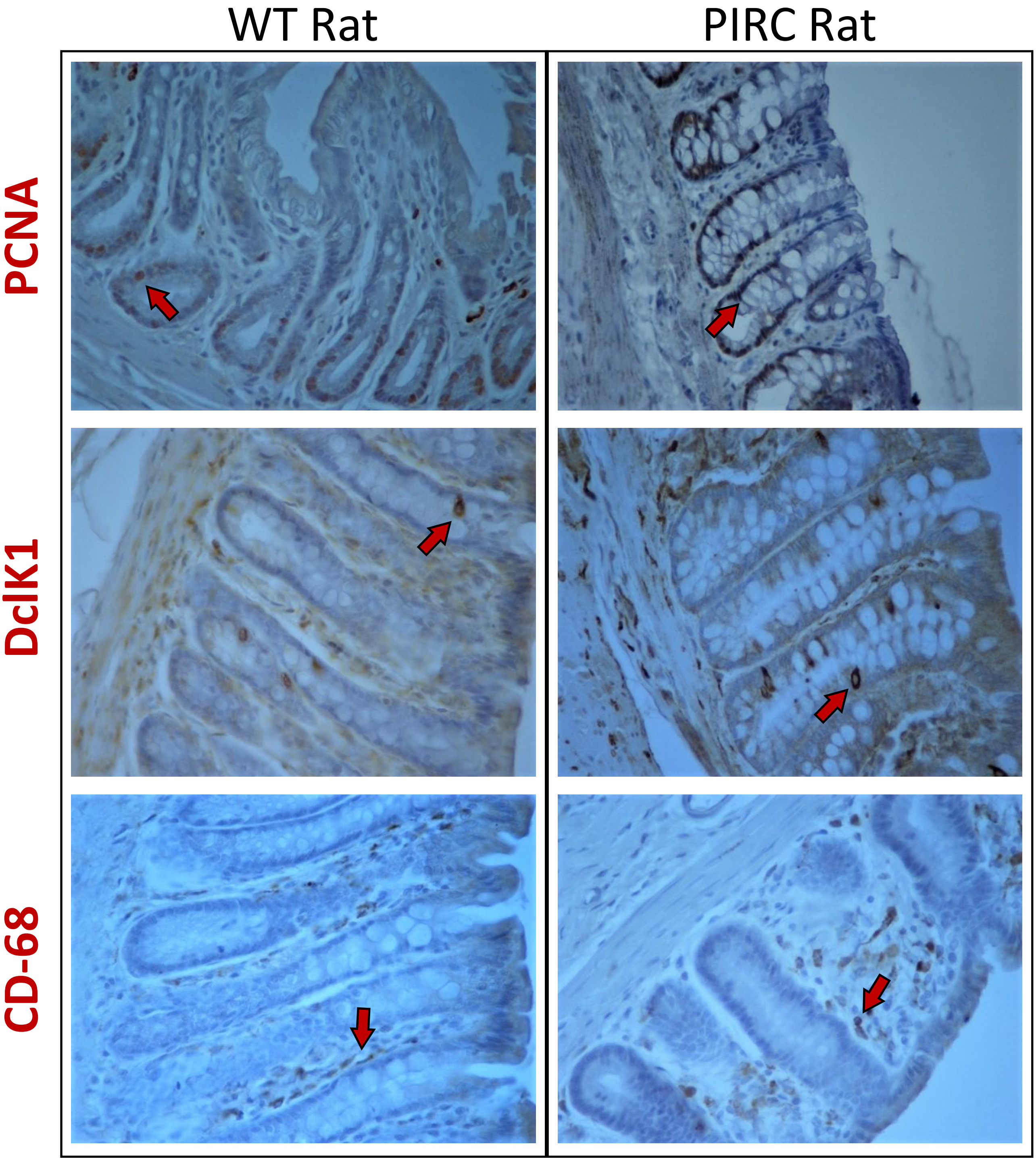

Supplementary Figure 5: Expression of PCNA, DclK1, and CD-68 in the normal mucosa of WT rats (left columns) and PIRC rats (right columns) as determined with immunohistochemistry experiments. Arrows indicate examples of positive cells. Original magnification 400x.
